# Mental health of agricultural adolescents and adults: Preliminary results of a five-year study

**DOI:** 10.3389/fpubh.2023.1056487

**Published:** 2023-03-02

**Authors:** Josie M. Rudolphi, Richard L. Berg

**Affiliations:** ^1^Department of Agricultural and Biological Engineering, University of Illinois, Urbana, IL, United States; ^2^Research Computing and Analytics, Marshfield Clinic Research Institute, Marshfield Clinic, Marshfield, WI, United States

**Keywords:** agriculture, adolescent, mental health, stress, depression

## Abstract

**Background:**

Work-related stressors common to agriculture have been associated with adverse mental health outcomes among adult farmers and ranchers. However, the mental health status of agricultural youth is unknown, despite farm and ranch youth being exposed to the same occupational hazards as their adult counterparts. The objective of this study was to estimate the prevalence of symptoms of depression and anxiety among farm adults and their adolescent child and examine the correlation between symptoms of mental health conditions and financial indicators described in the Family Stress Model (FSM).

**Methods:**

Farm families were recruited to participate in online surveys by mail, email, and social media. One adolescent and at least one adult from each family were invited to complete on online survey. Where available, validated instruments were used to collect mental health, stress, family dynamics, and household financial variables. Descriptive statistics were used to describe sample demographics and prevalence of symptoms of depression and anxiety. Pearson correlations describe associations between variables within the Family Stress Model.

**Results:**

Farm families (*N* = 122) completed the online survey. The mean age of farm parents was 41.4 years (SD = 4.4) and the mean age of farm adolescents was 15.4 (1.2). A majority of farm parents and farm adolescents were male, 58.2% and 70.5%, respectively. The sample was primarily white, non-Hispanic. In this sample of farm parents and adolescents alike, 60% met the criteria for at least mild depression, based on the Patient Health Questionnaire-9 (PHQ-9) and Patient Health Questionnaire-A (PHQ-A). Similarly, among adolescents, 45.1% met the criteria for Generalized Anxiety Disorder (GAD), as did 54.9% of adults. As a measure of economic hardship, per capita income by itself showed relatively low correlations, even with other economic measures (*r* = 0.11 with negative financial events, *r* = 0.20 with financial needs, *r* = 0.17 with financial situation, and *r* = 0.27 with debt). Parent depressed mood was in turn highly associated with adolescent depression (*r* = 0.83), social anxiety (*r* = 0.54), and generalized anxiety (*r* = 0.69).

**Conclusions:**

The results show a strong association between parent and adolescent mental health and parental depressed mood and debt. There is not a clear association between economic stress and mental health in this sample, but further work is needed to be done at a population level. Preliminary results are promising for application of the full Family Stress Model as we continue to accrue farm families into the study cohort.

## 1. Introduction

In the United States, sectors of the agricultural industry have been experiencing economic downturn in the last decade (1). Among farmers, personal finances, economics, and fluctuating commodity prices are consistently cited as sources of stress. In addition to financial factors, interpersonal relationships, farm succession, and unpredictable and unreliable environmental conditions, such as natural disasters and drought, also contribute to producers' stress (2–5). Chronic stress has been associated with a myriad of mental health outcomes such as anxiety, depression, and substance use. The prevalence of common mental health conditions, specifically anxiety and depression, is consistently higher among adult farmers than the general population (5–7).

The mental health of agricultural producers and workers has become a national priority. Cooperative Extension Services, United States Department of Agriculture (USDA), state departments of agriculture, non-profits, and health and safety professionals have organized efforts to address the growing concern. Research has estimated the prevalence of mental health conditions among the population and sub-populations (3, 5, 6, 8, 9). Mental health literacy programs have been developed and evaluated to increase self-efficacy among non-mental health professionals to respond to crises in rural and farm communities (10). Mental health providers have partnered with commodity groups and agricultural organizations to offer low or no-cost behavioral health services to agricultural (11). However, research, resources, and services have largely focused on adult agricultural producers.

Agriculture is a unique occupational industry. A majority of agricultural work occurs on private, independently owned and operated farms and ranches, which often also serve as a residence for children (1). As such, adolescents and children are often present in the agricultural environment, and in some instances, participating in agricultural work. Consequently, youth are exposed to the same work-related hazards of their adult counterparts, such as machinery and livestock.

Agricultural work contributes to injuries and illnesses among youth. There is a growing number of epidemiologic studies of injuries to farm children which describe risk factors, medical outcomes, and injury agents (12, 13). As a result, interventions have been evaluated and policies recommended to prevent child agricultural injuries (14–18). Similarly, there are recognized hazards in the agricultural environment, such as chemicals, gases, extreme climate conditions, physical hazards including noise, and zoonotic diseases which threaten the health of agricultural youth. While the work-related stressors and associated mental health outcomes among adult farmers are well-documented (5–7), and injuries to children well-examined (12, 13), the mental health of farm adolescents is unknown.

The main objective of this five-year project is to examine the relationships between work-related stress, family disruption, and mental health of farm adolescents and their farm parents by examining associations and relationships within the Family Stress Model (FSM). In this analysis, we describe the study population and identify the prevalence of positive screens for common mental health conditions among farm adolescents and adults and look for correlations between variables of the Family Stress Model. Data for this analysis was collected in summer, 2021.

### 1.1. Family Stress Model

The Family Stress Model suggests specific processes through which economic hardship is expected to impact adolescent development by way of family disruptions, relationships, and parenting. The FSM was originally conceptualized by Conger et al., who tested the adequacy of the model on a sample of Midwest, US, farm, and rural families experiencing economic hardship during what would come to be known as the 1980's Farm Crisis (19). Results supported the central hypothesis that coercive family processes may link family economic stress to problematic child and adolescent development (19).

The FSM [A schematic of the Family Stress Model can be found in Conger et al. (20)] suggests that economic hardship, characterized by negative economic events and coupled with low family income, increase the economic pressure experienced by parents. Negative economic events include a myriad of situations including job-loss, home foreclosure, forced sale of land, and alike. Economic pressure reflects the realities of economic hardships and include cutback on essential goods and services, daily expenditures, and forgoing bills. The FSM proposes economic pressure will increase emotional distress among parents, including symptoms of psychological distress, which will then disrupt the functioning of their relationship and intensify conflict in the relationship. Inter-parental conflict then disrupts effective parenting and strains the child–parent relationship and poor parenting will predict child emotional and behavioral problems (19–21). The FSM has been applied to various populations to examine the relationship between economic pressure, parental emotional and behavioral problems, and adolescent externalizing and internalizing behaviors (19–23).

## 2. Materials and methods

### 2.1. Study design

This cross-sectional study was conducted among agricultural producers and their adolescent children from June to August 2021 in the US.

### 2.2. Target population

The target population was farm adolescents between the ages of 13 and 18 years old and their parents residing on farms in the U.S. Five study states (Illinois, Iowa, Wisconsin, Minnesota, and Iowa) were selected because they are among the most agriculturally productive in the U.S., have a high proportion of primary producers that report farming as their full-time operation, and a high proportion of primary operators that reside on the farm they operate (24). However, during recruitment the target population was expanded to include farm and ranch families from the U.S.

### 2.3. Recruitment

Two primary strategies were employed to recruit farm families into the study.

#### 2.3.1. Agricultural producers list

A list of addresses of 1,000 agricultural producers in seven Midwestern states with at least one adolescent in the household was purchased from Farm Market iD (FMiD). FMiD maintains a list of farm owners and operators based on the same sources as the U.S. Department of Agriculture's (USDA) databases and estimates 95% coverage of farm owners and operators. FMiD overlayed USDA agricultural census variables with consumer variables to identify primary producers who work on a farm full-time, live on the same farm they operate, and have at least one adolescent (aged 13–17) in their household (25).

Recruitment and survey materials were mailed to the 1,000 agricultural producers using a modified Dillman approach to encourage survey response (26). Dillman encourages unique, repeated contacts with potential participants (26). An introductory postcard to pique interest was sent to each address. Three weeks later, a packet of information followed which included the objectives of the study, IRB information, directions for participating, and a QR code to the online surveys. Three weeks later, a reminder postcard was sent which included the QR code to the online surveys.

#### 2.3.2. Online study blog

Recruitment also occurred online. The project team partnered with Extension, commodity groups, and farm organizations to disseminate information about the study via email, newsletters, and social media. Emails, newsletters, and social media posts directed individuals to an online study blog that described the objectives of the study, provided IRB information, directions for participating, and a link to the online surveys. Two, duplicate online surveys, with unique web addresses, were created to distinguish participants by recruitment method.

### 2.4. Procedures

The project team worked with the Research Electronic Data Capture (REDCap) administrators at the University of Illinois Urbana-Champaign to develop a series of consent forms and assent forms in addition to the online surveys, to ensure protection of participants. REDCap is a secure, web-based application designed to support data capture for research studies, providing: (1) an intuitive interface for validated data entry; (2) audit trails for tracking data manipulation and export procedures; (3) automated export procedures for seamless data downloads to common statistical packages; and (4) procedures for importing data from external sources (27).

A QR code and link on the study blog, provided on recruitment materials (letters, emails, social media posts), led to an adult/parent consent form. The consent form collected name, email address, and signature of an adult/parent (P1). The form also consented an adolescent in the household to participate and collected the name and email address of the consented adolescent. Finally, the form inquired about a second adult in the household and asked for the name and email of the second adult (P2), if applicable. After consenting themselves, consenting the adolescent in the house, and providing the contact information for a second adult (if applicable and optional) the adult proceeded to the P1 online survey. REDCap auto emailed the adolescent a link to the assent form, which had to be completed by the adolescent prior to unlocking the adolescent survey. Similarly, REDCap auto-emailed the second adult in the household a link to their consent form and ultimately their online survey, the P2 survey. REDCap linked family consent forms, assent forms, and surveys. All survey participants were emailed an electronic Amazon gift card valued at $10.

### 2.5. Materials

Online surveys were used to collect information about farm economics, family dynamics, and mental health from farm parents and adolescents. Here, we report on all survey sections; however, not all are described in the results or discussion as sample size precluded statistical modeling of FSM constructs.

#### 2.5.1. Adult survey

The Farm Stress and Mental Health Adult Survey was completed by P1 and P2, if applicable. The adult survey took a median of 19 min to complete and could be completed on a smartphone, tablet, or computer. The adult survey included the following sections and questions and/or instruments.

##### 2.5.1.1. Screening

Adults were asked whether they identify as a (1) primary operator or principal operator of a farm, ranch, or agricultural operation, (2) partner or spouse of the primary operator or principal operator of a farm, ranch, or agricultural operation, or (3) none of the above. Adults were asked whether they reside, at least 50% of the time, on the farm, ranch, or agricultural operation. In order to proceed to the remained of the survey, adults must have identified as either the primary operator or partner/spouse of the primary operator and reside on the farm/ranch/agricultural operation at least 50% of the time.

##### 2.5.1.2. Demographic and farm characteristics

Adults responded to the following questions: state of residency, age, gender, ethnicity, race, education, material status, number of children had, age range of children, number of children in the household, military status, employment status, number of years farming, primary farm commodity, secondary farm commodity, annual farm/ranch net sales, and residential environment.

##### 2.5.1.3. Health

Adults responded to the following questions about their health: diagnosis of chronic health conditions (asthma, arthritis, anxiety, caner, COPD, dementia and/or Alzheimer's Disease, depression, diabetes, hearing loss, heart condition, high blood pressure, high cholesterol), weight, height, and average number of hours of sleep each night.

##### 2.5.1.4. Mental health

The Generalized Anxiety Disorder-7 item scale (GAD-7) was employed to identify self-reported symptoms of anxiety among adults. This seven-item questionnaire has been widely used in population-based survey studies (28) and among farm populations (5, 29) and has high internal validity (*a* = 0.81), sensitivity (89%), and specificity (82%) (28). Participants responded to seven unique statements that queried the frequency of anxiety-related symptoms as experienced in the past 2 weeks (e.g., *not being able to stop or control worrying*). Response options were; not at all, several days, over half the days, nearly every day. Response options were assigned a point-value; not at all = 0, several days = 1, over half the days = 2, nearly every day = 3. The response option point-values for each of the seven unique anxiety-related symptoms were totaled for an individual's total score. The possible score range is 0–21. Scores of 5–9 indicate mild anxiety, 10–14 moderate anxiety, and 15–21 severe anxiety (28).

The Patient Health Questionnaire-9 (PHQ-9) identified self-reported symptoms of depression. The nine-item questionnaire has been used to estimate the prevalence of depression among the general U.S. population (30) and farmers (5, 29) and demonstrates high internal validity (*a* = 0.91), sensitivity (88%), and specificity (89%) (Kroenke et al., 2001). Participants responded to the nine statements that inquire about the frequency of depressive symptoms as experienced in the past 2 weeks (i.e., poor appetite or overeating, feeling tired, or having little energy). Response options are; not at all, several days, over half the days, nearly every day. Each response option is assigned a point-value; not at all = 0, several days = 1, over half the days = 2, nearly every day = 3. The response option point-values for each of the seven unique depression-related symptoms are totaled for an individual's total PHQ-9 score. The possible score range is 0–27. Cut off points are: 0–4 is considered normal range or full remission; 5–9 is considered minimal depressive symptoms; 10–14 is considered major depression, mild severity; 15–19 is considered major depression, moderate severity; and 20 or higher is considered major depression, severe severity (30).

The Coronavirus Anxiety Scale (CAS) was used to identify probable cases of dysfunctional anxiety associated with the COVID-19 crisis. The five-item scale has demonstrated solid reliability and validity (31). Adults responded to each item indicating how often they had experienced each in the past 2 weeks on a five-point Likert scale (0 = not at all, 4 = nearly every day). Individual scores were generated by summing the five response options. The permissible range for scores is 0–20 with higher scores indicating more severe anxiety (31, 32).

##### 2.5.1.5. Interpersonal/Marital conflict

The Behavioral Affect Rating Scale (BARS) was used to assess relationship warmth and hostility among adult partners (33). Each adult responded to 22 items describing the behaviors of their partner over the past year. Adults indicated how often, on a Likert scale (1 = always, 4 = never), their partner engaged in 9 items related to warmth (affection, humor, and acted supportively) and 13 items related to hostility (shout/yell, insult). Items within each subscale (warmth and hostility) were summed. Higher scores indicate lower warmth and higher hostility. These scales have exhibited high predictive validity in prior studies (19, 22, 34).

##### 2.5.1.6. Social support

The Multidimensional Scale of Perceived Social Support (MSPSS) measured farm parents perceived social support (35) from three sources: family, friends, and a significant other. Participants responded with how strongly they agreed to 12 statements (e.g., *there is a special person who is around when I am in need*) on a seven-point Likert scale (very strongly disagree to very strongly agree). Each response was assigned a point value for a cumulative score. Three factor group (family, friends, and significant other) scores were calculated by summing the responses to each of the four items within each group with higher scores indicating more perceived social support (35).

##### 2.5.1.7. Parenting style

Parenting involvement and control were assessed using a modified version of the Steinberg instrument (36), a 15-item instrument that includes nine items related to involvement and six items related to control. Adults responded to each item indicating how much they agree with each item on a five-point Likert scale (1 = strongly disagree, 5 = strongly agree). A control and involvement score were calculated for each adult by summing the values of each response. Control and involvement scores were dichotomized at their median values to identify high and low control and involvement categories.

##### 2.5.1.8. Economic hardship

Adult respondents completed three subscales adopted from the economic pressure measures developed by Conger et al. (19): (1) make ends meet, (2) material needs, and (3) cutbacks/adjustments. The first subscale included two items indicating whether parents felt that they could “make ends meet” during the past 12 months. The first item was, “During the past 12 months, how much difficulty have you had paying your bills?” and response options were a great deal of difficulty, some difficulty, a little difficulty, and no difficulty at all. The second item was, “Think again over the last 12 months, generally, at the end of the month, do you end up with…” and response options were more than enough money left over, some money left over, just enough to make ends meet, not enough to make ends meet. The second subscale measured whether the household could meet its basic material needs. Adults responded to seven basic material needs (clothing, transportation, a home, furniture and household appliances, food, and medical services). Responses were dichotomously scored (1 = yes, 0 = no) and summed to create a subscale score. The third subscale assessed whether the family had made significant financial cutbacks in several areas, including food, medical care, and utilities, because of economic hardship. Responses were dichotomously scored (1 = yes, 0 = no) and summed to create a subscale score. Each indicator was coded so that higher scores reflected greater economic pressure.

##### 2.5.1.9. Stress

A modified Farm Stress Survey was used to identify sources of farm stress among adults. This 40-item instrument measures seven dimensions of stress (personal finances, time pressure, climate conditions, geographic isolation, hazardous working conditions, general economic conditions, and interpersonal relationships) (37). Participants indicated the extent to which each of the items of the Farm Stress Survey was a source of worry or concern in the past 2 weeks on a five-point Likert-type scale (1 = not at all, 5 = an overwhelming extent). *Farm Stress Survey* subscale scores were calculated by determining the mean response to the items within each subscale. Permissible subscale scores range from 1 to 5 give higher scores indicating higher perceived concern.

#### 2.5.2. Adolescent survey

The Farm Stress and Mental Health Adolescent Survey was completed by the farm adolescent. The adolescent survey took a median of 19 min to complete and could be completed on a smartphone, tablet, or computer. The adolescent survey included the following sections and questions and/or instruments.

##### 2.5.2.1. Screening

Adolescents were asked whether they reside, at least 50% of the time, on a farm, ranch, or agricultural operation. Adolescents were also asked their age, with the following response options to choose from: 13, 14, 15, 16, 17, 18, other. In order to proceed to the remainder of the survey, adolescents must have indicated they reside on a farm, ranch, or agricultural operation at least 50% of the time and indicated they were between the ages of 13 and 18. If an adolescent indicated they were not between the ages of 13 and 18 and/or did not reside on a farm or ranch or agricultural operation at least 50% of the time they were directed to the end of the survey.

##### 2.5.2.2. Demographics and farm characteristics

Adolescents responded to questions about age, school grade, race, state of residency, farm work status, hours per week involved in farm work during the summer and school year, farm injury experience.

##### 2.5.2.3. Health

Adolescents responded to the following questions about their health: self-rated physical and mental health, diagnosis of chronic health conditions (asthma, arthritis, anxiety, cancer, COPD, dementia and/or Alzheimer's Disease, depression, diabetes, hearing loss, heart condition, high blood pressure, high cholesterol), weight (reported in pounds), height (recorded in feet and inches), average number of hours of sleep each night.

##### 2.5.2.4. Mental health

The Patient Health Questionnaire-A (PHQ-A) identified self-reported symptoms of depression among adolescents. The PHQ-A is a modified PHQ-9 appropriate for adolescents and has demonstrated satisfactory sensitivity, specificity, diagnostic agreement, and overall diagnostic accuracy, compared with the clinical interview (38). Participants responded to the nine statements that inquired about the frequency of depressive symptoms in the past 2 weeks (i.e., *poor appetite or overeating, feeling tired or having little energy*). Response options are assigned a point-value; not at all = 0, several days = 1, over half the days = 2, nearly every day = 3. The response option point-values for each of the seven symptoms were totaled for an individual's total PHQ-A score. The possible score range is 0–27. Cut off points are: 0–4 is considered normal range or full remission; 5–9 is considered minimal depressive symptoms; 10–14 is considered major depression, mild severity; 15–19 is considered major depression, moderate severity; and 20 or higher is considered major depression, severe severity (38).

The Screen for Anxiety Related Disorders (SCARED) was used to screen farm adolescents for childhood anxiety disorders including general anxiety disorder, separation anxiety disorder, panic disorder, social anxiety, and school phobia. The SCARED consists of 41 items related to the 5 factors [general anxiety disorder (9 items), separation anxiety disorder (8 items), panic disorder (13 items), social anxiety (7 items), and school phobia (4 items)] that parallel the DSM-IV classification of anxiety disorders (39). Adolescents indicated how true each item was in the past 3 months on a three-point Likert scale (0 = not true or hardly true, 2 = very true or often true). Scores for each anxiety related disorder were calculated by summing the responses to the items within each factor. Cut-off scores indicating probable or likely anxiety vary by type of anxiety related disorder. Across all 41 items, a total score of ≥25 may indicate the presence of an anxiety disorder (39).

##### 2.5.2.5. Stress

The Adolescent Stress Questionnaire (ASQ) evaluated sources of stressors among adolescents. This is a 27-item inventory reflecting five dimensions of stress [home life (7 items), school attendance and teacher interactions (11 items), peer pressures (4 items), future uncertainty (3 items), financial pressure (2 items)]. Adolescents indicated how much stress each item has caused in the past 12 months on a Likert scale (1 = not at all, 5 = very stressful). Dimension scores were calculated by summing the affirmed response to each item within each dimension (40). The ASQ has been shown to be valid for measuring stress in research and clinical contexts (40, 41).

##### 2.5.2.6. Risk-taking behaviors

Adolescents self-reported risk-taking activities related to substance use, sexual activity, intentional/unintentional injury. Adolescents self-reported how often they participated in the following activities in the past 30 days and the past 12 months: number of times rode in a car or other vehicle driven by someone who had been drinking alcohol, seatbelt use when riding in a car driven by someone else, how many days they text or e-mail while driving a car or other vehicle, days carried a weapon, days carried a weapon on school property, days carried a gun, days skipped school, and number of times in a physical fight. Adolescents responded to whether they considered suicide, made a plan to attempt suicide, or had attempted suicide in the past 6 months (42). Adolescents indicated whether they ever tried a substance, how old they were when they first tried the substance, how many days they used the substance in the past 30 days. The following substances were inquired about: cigarettes, electronic vapor products, chewing tobacco, alcohol (includes beer, wine, wine coolers, and liquors), marijuana, synthetic marijuana, prescription medication for non-medication purposes (42).

##### 2.5.2.7. COVID-19 experience

The COVID-19 Adolescent Symptom and Psychological Experience (CASPE) was used to identify farm youths' experience with COVID-19 (43). Adolescents responded to how much and how the COVID-19 outbreak negatively and positively impacted their daily life and how stressful the COVID-19 disruptions were. Adolescents also indicated to what extent they had felt 19 emotions (e.g., anxious, happy, worried, distressed, calm) in the past 7 days because of the COVID-19 outbreak on a five-point scale (very slightly or not at all—extremely). Adolescents indicated their level of concern about the COVID-19 outbreak on 16 circumstances or situations (i.e., having to stay home, a family member might get sick, having enough to eat) on a five-point scale (very little or not at all—a great deal). Finally, adolescents responded to six emotions (relaxed, hopeful, anxious) indicating how *much more* they experience the emotion compared to before the pandemic on a five-point scale (not at all—a great deal).

### 2.6. Data cleaning

Surveys for analysis were first limited to those where appropriate consents were registered and surveys were completed for one adolescent paired with at least one adult/parent participant. Various procedures were used to try to ensure that surveys for analysis were limited to eligible participants who seriously attempted to complete the instruments. It was apparent early that online recruitment had been subject to multiple cyber-attacks, including software-based, automated completion of surveys (i.e., attacks by internet robots or “bots”). This was obvious for several reasons. First, within hours of posting the survey link publicly online, there were over 520 responses, an unlikely influx. Additionally, there were series of up to 20 consecutive surveys submitted with identical entries, including identical start and stop times. As soon as the research team was aware of the issue, within 2 h of the original online post, the REDCap administrator added an attention check question to all surveys. The question asked participants to select the color red from a list of colors listed below.

After data collection was complete, a series of recommended checks were employed to identify and exclude bot-generated responses from analysis (44). A number of strategies have been recommended to identify and mitigate research fraud. Among the most effective strategies are screening email addresses, screening open-ended responses, and monitoring the time and speed of survey completion. Moderately effective strategies include checking the eligibility of IP addresses and embedding attention check questions into the survey (45). Based on these recommendations and available information, a combination of electronic processing and manual review was then used to create 11 flags for suspicious forms (described below).

First, duplicate forms were removed from the dataset. As mentioned previously, series of up to 20 consecutive surveys were submitted with identical entries, including identical start and stop times. All duplicate time entries were reviewed, and all duplicate and suspect survey series were excluded. Duplicate surveys were further identified by electronic identification of duplicate parent or adolescent e-mail addresses, and through manual review of suspicious follow-up e-mail solicitations to the investigators.

From there, the research team employed a combination of electronic and manual processing to create a series of 11 flags to help identify suspicious survey respondents. Five flags were related to discrepancies between parent/adult and adolescent surveys and included (1) different state residency for adolescent and adult; (2) less than 15-year difference between adult and adolescent ages; (3) age of adolescent outside the range reported for youngest and oldest child; (4) inconsistent race (parents only white with adolescent only black, or the reverse); (5) Latino adolescent with both P1 and P2 not Latino. Two flags were related to improbable or incorrect responses on the surveys and included (6) failure to select “RED” on security question where instructed simply to “Pick RED” as the response; and (7) height outside CDC norms of 0.05–99.95%. Embedding attention check questions, requesting a specific response from survey participants, is a moderately effective strategy for detecting bot responses and identify risk of lower data quality, however, in time, bots can learn the correct answer to the attention check questions (44).

Additionally, based on review of completion times (median 19 min), surveys with unusually short times were also believed to be invalid or incomplete and flagged (8). Surveys completed in <8 min were excluded. Monitoring the speed of survey competition has also been recommended as a method to identify bots (45).

The last three flags were based on responses to open text responses and included (9) incoherent or inappropriate text for primary farm commodity; (10) commodity reported in singular, for example “Ostrich” or “Goose”; (11) commodities noted as associated with submissions dropped as clearly invalid (e.g., “POTATOES, TOMATOES” or “Corn, barley, oats”). In the course of these reviews, the few free-text fields on the survey were noted as particularly helpful in identifying bot-completed forms, which aligns with recommendations for best practices (44). However, the process is time-consuming. The principle investigator manually reviewed submitted text for farm commodities and “Other” responses, flagging suspicious forms for exclusion. Based on review of flag frequencies, the study team decided that all remaining surveys with two or more flags would be excluded from analysis.

### 2.7. Data analysis

In the final analysis cohort of 122 families (one adult parent and one adolescent), relatively few response items were missing. For example, individual items in the PHQ-9 were missed for at most 2/122 (1.6%) for the adult (P1) and for at most 3/122 (2.5%) of adolescents, while for both adult and adolescent five of the nine individual items showed no missing responses. To allow for some missed items, summary scores for each instrument were calculated whenever over half of items were completed, and the mean of completed items was used to scale the summary score to the same range as possible with no missing items.

Descriptive summaries were created to characterize the analysis cohort using standard descriptive statistics. Cronbach's alpha was calculated as a measure of internal consistency for the items in a summary score. Pearson correlations were calculated to evaluate the strength of associations among potential FSM model indicators. In this analysis, we present demographic characteristics, correlations between FSM model indicators, and mental health outcomes.

Analyses were completed using SAS^®^ version 9.4 (SAS Institute Inc.) statistical software.

### 2.8. Ethical approval

All procedures performed and materials used in this study, involving human participants, were approved by the University of Illinois Urbana-Champaign Institutional Review Board.

## 3. Results

A total of 2,463 potential farm-family survey submissions were recorded. After flagging responses as described above and limiting analysis to observations with two or less flags, the final sample consisted of 122 family participants. For this analysis, families include responses from one adult parent (P1) and one adolescent. Among the 122 family participants, only 25 observations (20%) included responses from a second adult (P2). Therefore, responses from the second adult are not included in this analysis.

Table 1 shows the demographic and health characteristics of the 122 farm parents. The mean age of parents was 41.4 (SD = 4.4), and just over half of parents identified as men. A majority of farm parents were white (84.4%) and almost a quarter (22.1%) were Hispanic. Highest educational status was split between high school diploma, associate's degree, and bachelor's degree. In total, 68.0% of adults indicated they are the primary operator on the farm/ranch and over 95% are full-time on the farm/ranch. The mean number of years farming was 13.3 (SD = 5.8), with 60.7% residing in rural areas and 36.9% residing in suburban areas.

**Table 1:** Demographics and health characteristics of farm parents (P1) (*N* = 122).

**Demographic**	***N* (%) or Mean (SD)**
Age	41.4 (4.4)
Sex	
Male	71 (58.2%)
Female	51 (41.8%)
Race	
White	103 (84.4%)
Black or African American	14 (11.5)
Other	5 (4.1%)
Hispanic	27 (22.1%)
Veteran	14 (11.8%)
Education (highest level)	
High school diploma	50 (42.7%)
Associate degree	37 (31.6%)
Bachelor's degree	26 (22.2%)
Master's degree or higher	4 (3.4%)
Marital status	
Married	117 (97.5%)
Divorced/separated	3 (2.5%)
Single	0 (0.0%)
Residence	
Rural	74 (60.7%)
Suburban	45 (36.9%)
Urban	3 (2.5%)
Primary farm/ranch operator (vs. partner or spouse)	83 (68.0%)
Farm full-time (vs. part-time)	114 (95.8%)
Mean number of children	1.4 (0.6)
Median years farming	13.3 (5.8)
Median farm income (US Dollars)	120,000.00
Mean farm income (US Dollars)	277,444.00
BMI	
Underweight	16 (13.1%)
Normal	57 (46.7%)
Overweight	39 (32.0%)
Obese	6 (4.9%)
NA (could not be calculated)	4 (3.3%)
Self-rated physical health	
Excellent	38 (31.1%)
Good	62 (50.8%)
Fair	22 (18.0%)
Poor	0 (0.0%)
Self-rated mental health	
Excellent	42 (34.7%)
Good	61 (50.4%)
Fair	17 (14.0%)
Poor	1 (0.8%)
Chronic health condition	
Asthma	3 (2.5%)
Arthritis	20 (16.4%)
Anxiety	20 (16.4%)
Cancer	0 (0.0%)
COPD	3 (2.5%)
Dementia	0 (0.0%)
Depression	5 (4.1%)
Diabetes	3 (2.5%)
Hearing loss	3 (2.5%)
Heart condition	2 (1.6%)
Hypertension	25 (20.5%)
High cholesterol	2 (1.6%)

Among adults, 81.9% self-rated their physical health as excellent or good and 85.1% self-rated their mental health as excellent or good. The most common chronic health conditions diagnosed were arthritis (16.4%), anxiety (16.4%), and hypertension (20.5%).

As shown in Table 2, the mean age of farm adolescents was 15.4 (SD = 1.2), 70.5% were male, 83.5% were white, and 11.5% were Hispanic. Adolescents' educational levels ranged from 7^th^ to 12^th^ grade, with nearly three-quarters between 9^th^ and 11^th^ grade. Over half (53.7%) indicated they participate in farm/ranch work, working an average of 14.0 (SD = 10.3) hours a week during the school year and 19.7 (SD = 16.2) hours during school vacations (summer). A majority work on farm/ranch that is operated by their parents.

**Table 2:** Demographics of farm adolescents (*N* = 122).

**Demographic**	***N* (%) or Mean (SD)**
Age	15.4 (1.2)
Sex	
Male	86 (70.5%)
Female	36 (29.5%)
Race	
White	102 (83.6%)
Black or African American	14 (11.5%)
Other	
Hispanic	20 (16.5%)
Education level (current)	
7^th^	8 (6.6%)
8^th^	17 (13.9%)
9^th^	35 (28.7%)
10^th^	28 (23.0%)
11^th^	26 (21.3%)
12th	8 (6.6%)
Participate in agricultural work	65 (53.7%)
Mean hours of agricultural work during school year	14.0 (10.4)
Mean hours of agricultural work during non-school year	19.7 (16.2)
Operator of agricultural operation youth works on	
Parents	59 (92.2%)
Grandparents	3 (4.7%)
A neighbor, non-relative	1 (1.6%)
Other relative	1 (1.6%)
BMI	
Underweight	31 (25.4%)
Normal	67 (54.9%)
Overweight	14 (11.5%)
Obese	6 (4.9%)
NA (could not be calculated)	4 (3.3%)
Self-rated physical health	
Excellent	50 (42.0%)
Good	61 (51.3%)
Fair	8 (6.7%)
Poor	0 (0.0%)
Self-rated mental health	
Excellent	43 (35.5%)
Good	57 (47.1%)
Fair	18 (14.9%)
Poor	3 (2.5%)
Chronic health condition	
Asthma	5 (4.1%)
Arthritis	1 (0.8%)
Anxiety	23 (18.9%)
Cancer	0 (0.0%)
COPD	2 (1.6%)
Dementia	0 (0.0%)
Depression	8 (6.6%)
Diabetes	0 (0.0%)
Hearing loss	0 (0.0%)
Heart condition	0 (0.0%)
Hypertension	2 (1.6%)
High cholesterol	1 (0.8%)

Among farm adolescents, 93.3% self-rated their physical health as excellent or good whereas 82.6% self-rated their mental health as excellent or good. The most common chronic health condition diagnoses were anxiety (18.9%), depression (6.6%), and asthma (4.1%).

Among farm parents, 60.1% met the criteria for at least mild depression based on the PHQ-9 screening instrument (Table 3). Almost a quarter (24.6%) met the criteria for mild depression, 23.8% met the criteria for moderate depression, and 11.5% met the criteria for moderately severe depression. Similarly, 54.9% of farm parents met the criteria for at least mild generalized anxiety disorder (GAD). Nearly a third (31.3%) met the criteria for mild GAD, 18.9% met the criteria for moderate GAD, and 4.9% met the criteria for severe GAD.

**Table 3:** Symptoms and severity of depression and anxiety among farm parents (*N* = 122).

	**Farm parents (P1)**
	***N*** **(%)**
**PHQ-9**	
None (0–4)	48 (39.3%)
Mild (5–9)	30 (24.6%)
Moderate (10–14)	29 (23.8%)
Moderately severe (15–19)	14 (11.5%)
Severe (20+)	1 (0.8%)
**GAD-7**	
None (0–4)	55 (45.1%)
Mild (5–9)	38 (31.1%)
Moderate (10–14)	23 (18.9%)
Severe (15+)	6 (4.9%)

Table 4 shows the prevalence of symptoms of depression among farm adolescents and 60.7% met the criteria for at least mild depression based on the PHQ-A. Over a quarter (26.2%) met the criteria for mild depression, 22.1% met the criteria for moderate depression, and 11.5% met the criteria for moderately severe depression. Over two-thirds (67.2%) of adolescents met the criteria for separation anxiety disorder, 60.7% met the criteria for panic disorder, and half (50%) met the criteria for school avoidance (Table 5).

**Table 4:** Prevalence of symptoms of depression among farm adolescents (*N* = 122).

	**Farm adolescents**
	***N*** **(%)**
**PHQ-A category and score**	
None (0–4)	48 (39.3%)
Mild (5–9)	32 (26.2%)
Moderate (10–14)	27 (22.1%)
Moderately severe (15–19)	14 (11.5%)
Severe (20+)	1 (0.8%)

**Table 5:** Prevalence of symptoms of anxiety related disorders among farm adolescents (*N* = 122).

**Anxiety related disorder**	***N* (%) meeting cut-off criteria^a^**
Panic disorder or significant somatic symptoms	74 (60.7%)
Generalized anxiety disorder	55 (45.1%)
Separation anxiety disorder	82 (67.2%)
Social anxiety disorder	47 (38.5%)
School avoidance	61 (50.0%)

a Cut-off scores for probable anxiety vary depending on type of anxiety disorder.

Correlations among proposed indicators for the FSM are shown in Table 6. For this analysis, all indicators have been scaled in a similar direction (22), and so these correlations are expected to be between 0 and 1. As a measure of economic hardship, per capita income by itself showed relatively low correlations, even with other economic measures (*r* = 0.11 with negative financial events, *r* = 0.20 with financial needs, *r* = 0.17 with financial situation, and *r* = 0.27 with debt). The economic measure most associated with parent depressed mood was debt (*r* = 0.67). Parent depressed mood was in turn highly associated with adolescent depression (*r* = 0.83), social anxiety (*r* = 0.54), and generalized anxiety (*r* = 0.69).

**Table 6:** Matrix of Pearson correlation coefficients among indicators of the Family Stress Model (*N* = 122).

	**2**	**3a**	**3b**	**3c**	**4**	**5a**	**5b**	**6a**	**6b**	**7a**	**7b**	**7c**
1. Per capita income	0.11	0.20^*^	0.17	0.27^**^	0.25^**^	0.22^*^	0.24^**^	0.18^*^	0.14	0.08	−0.05	0.13
2. Neg financial events		0.82^**^	0.55^**^	0.45^**^	0.37^**^	0.35^**^	0.23^**^	0.36^**^	0.27^**^	0.29^**^	0.32^**^	0.37^**^
3a. Economics–financial need			0.52^**^	0.46^**^	0.39^**^	0.33^**^	0.25^**^	0.26^**^	0.17^**^	0.25^**^	0.30^**^	0.38^**^
3b. Economics–financial situation				0.56^**^	0.44^**^	0.41^**^	0.50^**^	0.41^**^	0.35^**^	0.30^**^	0.24^**^	0.37^**^
3c. Economics–debts					0.67^**^	0.58^**^	0.44^**^	0.54^**^	0.45^**^	0.60^**^	0.47^**^	0.59^**^
4. Parent depressed mood						0.65^**^	0.48^**^	0.49^**^	0.37^**^	0.83^**^	0.54^**^	0.69^**^
5a. Caregiver conflict hostility							0.29^**^	0.70^**^	0.57^**^	0.60^**^	0.46^**^	0.53^**^
5b. Caregiver conflict warmth								0.28^**^	0.25^**^	0.40^**^	0.13	0.27^**^
6a. Parenting style involvement									0.84^**^	0.44^**^	0.28^**^	0.37^**^
6b. Parenting style strictness										0.34^**^	0.20^*^	0.26^**^
7a. Adolescent: depressed mood											0.55^**^	0.67^**^
7b. Adolescent: social anxiety												0.75^**^
7c. Adolescent: generalized anxiety												1.0

^*^p ≤ 0.05, ^**^p ≤ 0.01.

## 4. Discussion and conclusions

Farm families play a critical role in ensuring a safe and adequate food supply. According to the United States Department of Agriculture's 2017 Agricultural Census, over 85% of all farms and just over 60% of land are family held, meaning owned and operated by individual or family units (1). Ensuring the health and safety of agricultural producers in the United States and worldwide is an international priority. Economic and environmental conditions have threatened the mental health of agricultural producers, who experience higher prevalence of anxiety, depression, and higher rates of suicide than some sectors of the general population (5, 29, 46). While youth are known to live and work on farms (1), limited research has examined the relationship between farm stress, parental mental health, and adolescent mental health. This five-year study will offer updated information about the shared experience of farm stress and mental health within farm families.

In this sample of 122 farm families, we observed a high prevalence of farm adults that were experiencing at least mild symptoms of anxiety and depression. Among adults, nearly a quarter met the criteria for mild depression and moderate depression and 11.5% met the criteria for moderately severe depression. While the PHQ-9 was used to screen for depression, and not diagnose, the observed distribution of respondents experiencing symptoms of at least moderate depression exceeds that of the general population. According to results from the National Health and Nutrition Examination Survey (NHANES), 8.5% of adults had depressive symptoms in 2019, characterized as a score of 10 or greater on the PHQ-9 in 2019. This statistic increased to 27.8% during the COVID-19 pandemic (March–April 2020) (47). It is important to note for comparison that farm family data was collected in the summer of 2021. For additional comparison, though the instruments to assess for depressive symptoms differ, according to the 2020 National Survey on Drug Use and Health (NSDUH), 8.4% of adults aged 18 and older experienced at least one major depressive episode in the last 12 months, characterized by “a period of at least 2 weeks when a person experienced a depressed mood or loss of interest or pleasure in daily activities, and had a majority of specified symptoms, such as problems with sleep, eating, energy, concentration, or self-worth” (48).

Nearly a third of adult participants in our sample met the criteria for mild Generalized Anxiety Disorder, and 18.9% and 4.9% met the criteria for moderate and severe GAD, respectively. Again, the GAD-7 was used to screen for GAD and not diagnose. Among the general population, in 2019, 3.4%, and 2.7% of adults experienced moderate or severe symptoms of anxiety, respectively (49). Among farm adults in our study, only 45.1% experienced no or minimal symptoms of anxiety, whereas among the general population, 84.4% experienced no or minimal symptoms in 2019 (49). More recent comparisons of the adult, general population in the United States from national public health datasets are not available. The prevalence of symptoms of depression and anxiety was higher among adult farmers in our study than the general population; however, the distribution of respondent by depressive category is consistent with what has been previously reported among Midwestern farmers and young adult farmers and ranchers (5, 9). The results from this study provide further evidence that agricultural producers experience depressive symptoms more than the general population and additional research and interventions are needed to identify services and resources for farmers and ranchers.

Among farm adolescents in our study, 22.1% met the criteria for moderate depression, 11.5% met the criteria for moderately severe depression, and 0.8% met the criteria for severe depression, based on the PHQ-A. In the U.S. it is estimated 4.1 million adolescents (aged 12–17), or 17.0%, experience at least one major depressive episode per year (48). Among the general population, adolescent females and adolescents reporting two or more races were more likely to experience a major depressive episode. The prevalence of farm adolescents meeting the criteria for moderate and moderately severe depression is high when compared to the general population, especially when considering the sample was largely male and largely Caucasian, which are not demographics considered at high risk of depression. However, direct comparisons cannot be made given different sampling strategies, timing, and instruments. Nearly half (45.1%) of farm adolescents in our study met the criteria for generalized anxiety disorder and 67.2% met the criteria for separation anxiety disorder (50). These statistics are higher than what is observed in the general population, where an estimated 31.9% of adolescents show any anxiety disorder; however, direct comparisons are difficult to make, and reliable comparisons are dated. Although resources and services to serve adolescents and youth have increased, it remains unclear if resources and services specific to farm adolescents are available and disseminated in rural areas.

Statistics from national public health surveys suggest a mental health crisis in the U.S. (48). Conversations around mental health in the U.S. have increased in response to economic conditions and the COVID-19 pandemic as many Americans experienced isolation, uncertainty, and financial pressure (51). Farm adolescents and farm adults in our sample reported symptoms of anxiety and depression at relatively high rates. Importantly, many Americans experience barriers to mental health care such as cost, access, and stigma (52), and the issues may be exacerbated in rural communities, where services are often limited. Without appropriate intervention, these symptoms in farm adolescents may indicate risk for more serious mental health issues in the future. Also, if not addressed, these issues reported by farm youth may add to the challenges youth face in continuing their family traditions of careers in farming and ranching.

Among our sample of farm adults 34.7% and 50.4% self-rated their mental health as excellent and good, respectively. Similarly, among farm adolescents, 35.5% and 47.1% self-rated their mental health as excellent and good, respectively. Single item, self-rated mental health (SRMH) measures to assess mental health are increasingly popular in epidemiological surveys, because they reduce respondent burden and simplify analyses (53). Some analyses have demonstrated moderate correlation between SRHM and various mental health scales, including the PHQ-9 (54, 55). However, when considering our sample of adults, 85.1% rated their mental health as excellent or good and at the same time, 60.1% met the criteria for at least mild depressions, and 54.9% met the criteria for at least mild anxiety. These conflicting results suggest disagreement between self-rated and symptom-based mental health measures among the farmers. While validating single-item measures is beyond the scope of this study, there are important implications for farmer mental health. Poor or fair SRMH has been associated with increased healthcare utilization (56, 57). Therefore, the perception of excellent or good mental health, when clinical disorders may be present, may preclude farmers from seeking and obtaining mental health care, delaying treatment, and potentially reducing overall quality of life.

Results from this study show relatively low correlation between per capita income and negative financial events, financial needs, financial situation, and debt. Importantly, parental depressed mood was significantly correlated with all economic indicators, income, and debt. In addition, and perhaps one of the most important result of this analysis, the correlation between parental depressed mood and adolescent depressed mood was high (0.83). These results are very consistent with previous applications of the Family Stress Model and suggest there is a relationship between emotional distress and financial and economic situations among both farm parents and farm adolescents. This relationship has been documented in previous research of non-farm families (58, 59). The small sample size (*N* = 122) precluded the research team from further statistical modeling in line with the Family Stress Model, however, this is an area for future analysis in future years.

While preliminary results contribute to the body of knowledge around farm family mental health, there are limitations to consider. The small sample size precluded full statistical modeling of the Family Stress Model as demonstrated in previous applications of the theory (19, 21–23). However, in the future, pooling responses from several years is expected to provide more stable estimates and an opportunity for this statistical approach. Additionally, the relatively small sample size and the single point-in-time assessment threaten the generalizability of results. Mailed recruitment efforts yielded very low responses from farm families (*n* = 18) which encouraged the research team to consider online recruitment efforts, including using social media. Online recruitment resulted in a compromised survey link and thousands of questionable survey responses. While the research team employed evidence-based best practices for identifying illegitimate responses (44, 45), this is certainly a limitation of the data. However, recruitment of farm families into research is challenging given no centralized location and geographical distribution. Online efforts, including the use of social media, can reach a large number of people at a relatively low cost (60). Parents were required to consent their adolescent to participate in the study, and parents were informed the adolescent survey would inquire about stress, mental health, and substance use, which could have introduced bias among adolescents who did not want to respond to items truthfully. To reduce this bias, adolescent surveys were sent directly to an adolescent's email and they were encouraged to take the survey in a quiet, private location free from distractions. We also emphasized that survey results were anonymous, meaning we could not link responses to a specific person. Social desirability bias is often diminished when self-administered surveys are employed (61). Here, as with all surveys, uncertainty is introduced by missing items which may have been selectively skipped, but rates of missing responses were relatively low. Finally, comparisons of symptoms of depression and anxiety between farm and non-farm adults and adolescents should be considered with caution. The year, methodologies, and instruments used in data collection differed and direct comparisons could not be made.

To our knowledge, this is the first published study to examine the correlation between farm parent and adolescent mental health since the Conger et al., initial study of farm and rural families in Iowa (62). The results here, when considered with what is known, underscore the need for continued research including surveillance and interventions related to the mental health of farm families. A major strength of this study, as it continues, will be the application of the Family Stress Model, which has been well-examined and applied to various audiences including farm and rural families. The FSM remains a useful framework for conceptualizing the impact of economic pressure on farm adult and adolescent mental health, especially when considering economic challenges associated with farming remain a consistent and leading stressor among agricultural producers (2, 5, 6, 8, 63). The use of validated instruments to assess economic hardship, parenting styles and relationships, adult mental health, and adolescent mental health further strengthen the study.

Adolescent mental health is a public health priority, and increased attention on agricultural producer mental health has led to a needed influx of research, resources, and services to meet the unique need of the occupation. This research contributes important information about the prevalence of symptoms of anxiety and depression among a sample of farm parents and adolescents and correlations within families for consideration of resource and service development. Importantly, farm parent and adolescent mental health, specifically, symptoms of depression and anxiety are highly correlated. Additional research is required to further understand these relationships and the risk and protective factors for adolescent and adult mental health. Additionally, mental health resource and services should consider the family and influence of farm economics on farm family mental health.

## Data availability statement

The raw data supporting the conclusions of this article will be made available by the authors, upon request, when the funding cycle is completed.

## Ethics statement

The studies involving human participants were reviewed and approved by University of Illinois Urbana-Champaign Institutional Review Board. Written informed consent to participate in this study was provided by the participants' legal guardian/next of kin.

## Author contributions

JR and RB conceptualized the study. JR collected data. RB conducted data analysis. All authors contributed to the article and approved the submitted version.
